# Targeting Platelet Activation Pathways to Limit Tumour Progression: Current State of Affairs

**DOI:** 10.3390/ph15121532

**Published:** 2022-12-09

**Authors:** Kutlwano R. Xulu, Tanya N. Augustine

**Affiliations:** School of Anatomical Sciences, Faculty of Health Sciences, University of the Witwatersrand, 7 York Road, Parktown, Johannesburg 2193, South Africa

**Keywords:** thromboxane, ADP, thrombin, Aspirin, Clopidogrel, Atopaxar, cancer, platelet

## Abstract

The association between cancer and a hypercoagulatory environment is well described. Thrombotic complications serve not only as a major mortality risk but the underlying molecular structure and function play significant roles in enhancing tumour progression, which is defined as the tumour’s capacity to survive, invade and metastasise, amongst other hallmarks of the disease. The use of anticoagulant or antiplatelet drugs in cardiovascular disease lessens thrombotic effects, but the consequences on tumour progression require interrogation. Therefore, this review considered developments in the management of platelet activation pathways (thromboxane, ADP and thrombin), focusing on the use of Aspirin, Clopidogrel and Atopaxar, and their potential impacts on tumour progression. Published data suggested a cautionary tale in ensuring we adequately investigate not only drug–drug interactions but also those unforeseen reciprocal interactions between drugs and their targets within the tumour microenvironment that may act as selective pressures, enhancing tumour survival and progression.

## 1. Introduction

The earliest documented association between cancer and the vascular system was by the surgeon Sushruta, circa 1000 BC [1,2], in which tumour entry into the blood vessels was described as resulting in vessel compression and constriction [3]. Much later, in his 1865 lecture, Armand Trousseau proposed a relationship between thrombophlebitis and the underlying presentation of gastric cancer. These findings were echoed by Osler and McCrae in 1900 and Sproul in 1938 [3], setting the stage for decades of research investigating the relationship between thrombotic events and tumour progression. Cancer incidence is rising, particularly in sub-Saharan Africa [4]. Breast cancer, the most commonly diagnosed cancer in women worldwide, with an estimated 2.3 million new cases in 2020, is the fifth leading cause of cancer-related mortality [4,5]. In 2020, breast cancer accounted for 11.7% of new cases, followed closely by cancers of the lung (11.4%), prostate (7.3%), skin (6.2%), colon (6%) and stomach (5.6%) [4]. In males, lung cancer remains the most commonly diagnosed and leading cause of cancer-related mortality [4]. It is estimated that the incidence of breast cancer will remain high in females, whereas prostate cancer will become a leading cause of death in males [6].

Cancer is a heterogeneous disease. Differences observed within a single tumour, termed intratumoral heterogeneity [7,8], determine the risk for tumour survival and progression parameters, such as angiogenesis, invasion and metastasis [9]. These differences are driven by evolutionary principles described by cancer stem cell theory and clonal evolution theory [10], which are yet to be completely unravelled given the complexity of the tumour microenvironment (TME) and selective pressures exerted by non-tumour cells. The difference between tumours, termed intertumoral heterogeneity, may be classified according to histopathological presentation [8,9,11]. Tumour heterogeneity describes vast differences within and between tumours that are reflected by molecular subtypes and unique genetic, epigenetic and immune landscapes. Thus, the genomic properties of each tumour provide a deeper description of the characteristics underwritten by tumour processes and ultimately inform treatment strategies [12,13,14]. Cancer-associated thrombosis is a notable cause of morbidity and mortality. Patients presenting with cancer are six times more likely to develop venous thromboembolism (VTE), including pulmonary emboli and deep vein thrombosis, while arterial events could include stroke and myocardial infarction [2]. Thromboprophylaxis was suggested as a mechanism to reduce thromboembolic events; however, there remains limited uptake in the clinical setting [1,2].

In understanding the biology that underwrites the relationship between vascular events and cancer, it is becoming increasingly evident that dysregulation of vascular homeostasis facilitates tumour progression [15]. In this review, we discuss the underlying pathophysiology of cancer-associated thrombosis by defining reciprocal interactions between tumour cells and platelets within the TME. We explore the molecular mechanisms of selected anticoagulant/antiplatelet drugs specifically on platelet activation pathways and the implications for tumour progression that can be described as a tumour’s capacity for survival and metastasis.

## 2. Pathophysiology of Thrombotic Events: The Role of Platelets

### 2.1. Thromboembolic Events

**Figure 1:**
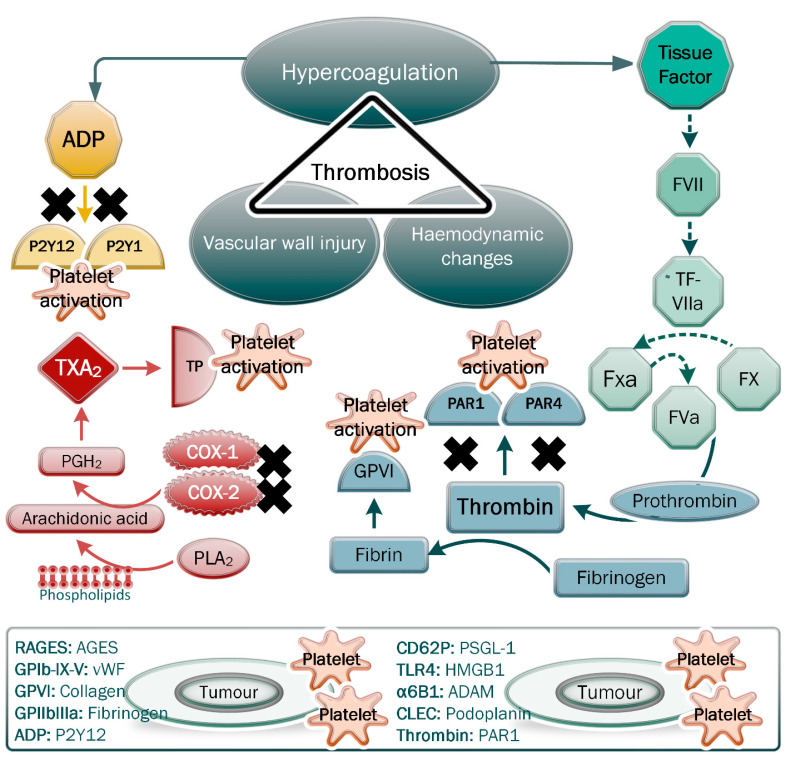
Virchow’s triad showing the main parameters of thrombosis. Regarding hypercoagulation, note the effects of the secreted platelet agonist tissue factor, ADP, thrombin and thromboxane A_2_ on the pathways inducing platelet activation and fibrin formation. Tumour cells are able to secrete these platelet agonists, which, following induction of platelet activation, cause the release of microparticles and a host of factors that can facilitate tumour progression and thrombotic complications. The direct and indirect interactions between platelet receptors/ligands and cognate ligands/receptors on tumour cells are shown. The inhibition of PAR1 and PAR4 receptors, the P2Y12 and P2Y1 receptors, and the production of thromboxane A_2_ (TXA_2_) are indicated as mechanisms that prevent platelet activation and downstream effects.

In cancer patients, the thrombotic state is characterised by reciprocal interactions between endothelial dysfunction, blood flow stasis and hypercoagulation, which is known as Virchow’s Triad (Figure 1) [2]. Thrombosis commonly represents the earliest clinical symptom of cancer and may be further promoted by the effects of surgery, chemotherapy, radiotherapy, long-term bed rest or trauma [16]. Thrombosis is associated with poor prognosis, thus reducing short-term and long-term survival in cancer patients [17,18].

Arterial thrombosis typically leads to myocardial infarction and stroke, whereas venous thrombosis is associated with venous thromboembolism (VTE) and pulmonary embolism (PE) [17,19]. Phenotypically, arterial thromboses consist of more platelets and occur at a site of injury where there is a high shear flow [18]. In contrast, venous thromboses, in addition to platelet involvement, consist of fibrin networks in low shear flow regions that may occur independently of vessel injury [17,19]. Cancer patients are inherently pre-disposed to the development of VTE, with approximately 15–32% of patients affected, contrasting with an approximately 2.5% incidence in non-cancer patients [20,21]. Moreover, VTE is more aggressive in cancer patients and does not respond well to therapy against it; therefore, it recurs [20,21]. VTE risk differs according to the tumour type; breast cancer is associated with a lower risk for VTE compared with other cancer types, such as pancreatic, colorectal and prostate cancers [2,18,20]. Despite this relatively low risk, hypercoagulation is increasingly being associated with tumour progression, failure of treatment and recurrence in breast cancer [2,18,20]. Clinically, VTE risk can be described using circulating D-dimer, thrombin–antithrombin, tissue factor (TF) and fibrinogen, as well as pro-thrombin time and activated partial thromboplastin time [22], many of which are predicated on molecular events that are a result of platelet activation, which is a critical step in facilitating hypercoagulation and associated events.

### 2.2. Platelets in Hypercoagulation

Platelets are key mediators in vascular homeostasis; furthermore, they play major roles in immunity and inflammation. Their role in vascular injury is via the processes of activation, adhesion and aggregation, thereby forming a clot to prevent bleeding and promote healing [3]. These small (~2 µm diameter), anucleate cytoplasmic fragments are primarily released from megakaryocytes in the bone marrow. An estimated production of 100 billion platelets is required daily to maintain a normal range [23]. In pathophysiological conditions, such as cancer, these normal processes are dysregulated, resulting in a hypercoagulable environment that instead facilitates tumour progression and concurrently creates thrombotic risk. Tumour-bearing patients often present with elevated levels of circulating platelets. Thrombocytosis defines an increase in the number of platelets, although the risk thereof differs with each cancer type. Thrombocytosis is associated with poorer outcomes, but the clinical utility of this parameter as a biomarker remains unclear, although it was noted that thrombocytosis can substantially precede the clinical detection of tumours [24,25,26]. Such secondary thrombocytosis in clinically detectable tumours and the presence of D-dimer is associated with VTE risk [22]. The number of thrombocytosis cases was shown to be high in patients with tumours of the kidney, oesophagus–gastric tube, breast, bladder and pancreas [25]. Thrombocytosis is associated with worse prognosis in a range of tumours, including lung, colorectal, gastric, kidney, brain, pancreatic, gynaecologic tumours and breast cancer [25]. The exact biological factors that account for these differences have not yet been defined but are suggested to be associated with the process of tumorigenesis in each site [25], with more research required into the precise role of platelets in mitigating or enhancing tumour processes.

Platelets present with a range of integrins and glycoproteins that mediate adhesion, as well as coagulation factors that drive a positive feedback loop that enhances platelet activation and the coagulation cascade to stabilise the developing thrombus [3]. Adenosine diphosphate (ADP), TXA_2_ and thrombin are agonists that drive platelet activation, leading to the expression of surface glycoproteins that facilitate adhesion [27]. These glycoproteins include GPVI and GPIbα, which bind to subendothelial extracellular matrix (ECM) proteins, collagen and von Willebrand factor (vWF), respectively, with GPIIb/IIIa also being essential for platelet activation and aggregation [28]. Coupled with agonist activity, ligation of these receptors results in dynamic morphological changes that increase the platelet surface area for adhesion and permit the secretion of granular contents, including growth factors, cytokines and chemokines [27].

There are three main types of granules in platelets, namely, α-granules, dense granules and lysosomes, which vary in terms of their formation, cargo and secretory kinetics [27,29]. α-Granules are specific to platelets and are the most abundant (50–80) [27,29]. The diverse repertoire of proteins contained within these granules includes coagulation factors (e.g., factors V, XI and XII), adhesion molecules (e.g., fibrinogen and vWF), molecules involved in immunoregulation (e.g., immunoglobulins and complement), cytokines (e.g., chemokines platelet factor IV and β-thromboglobulin) and growth factors (e.g., TGF-β, IGF, FGF and VEGF) [30].

Not all proteins are synthesized and packaged within megakaryocytes prior to budding off the proplatelet and subsequent maturation. Some proteins are endocytosed from plasma either via receptor-mediated or receptor-independent pathways [30]. As such, proteins of non-platelet origin can be taken up into platelets and released upon tumour-induced platelet activation [31]. In addition, platelets contain cytosolic mRNA derived from megakaryocytes and are thus able to synthesize new proteins. Platelets further contain miRNAs that can also alter protein translation [15,32]. Some authors put forward the complexity of platelet α-granules as heterogeneous with functionally distinct subtypes, while other authors suggest they are homogeneous but subject to variation in their response to agonists and their secretory dynamics [33,34,35,36]. This concept becomes important in the TME, where an understanding of tumour-secreted coagulation factors that activate platelets to later allow a platelet-facilitated epithelial–mesenchymal transition (EMT) and metastasis is needed.

Tumour-secreted thrombin activates platelets via PAR1 and PAR4 receptors, promoting the release of intracellular Ca^2+^ stores to enhance platelet activation and granule exocytosis. Notably, under thrombin stimulation, α-granules rapidly release their contents, with dense granules secreting their contents afterwards. Dense granules are the second most common granule within platelets and contain calcium, ADP, ATP and serotonin that enhance platelet activation and aggregation [30]. While granule secretion is dependent on the release of Ca^2+^ stores, the small GTPase known as Ral was implicated in regulating dense granule exocytosis, even in sub-maximally activated platelets [37]. That there is a range of platelet activation has become increasingly evident, with studies indicating that agonist concentration and type, for example, collagen-mediated ligation of GPVI, can induce granular secretion independent of major structural alterations [38]. Traditional coagulation studies, such as platelet aggregometry [39], are not able to detect the nuances involved with such shifts in secretory phenotype. Flow cytometry is suggested as a tool for assessing multiple platelet parameters to aid in understanding the process of platelet activation [40] and to show the gradation of platelet activation.

Flow cytometric assessment of, for example, CD62P (p-selectin), which is contained within α-granules and translocates to the platelet membrane surface during early activation and degranulation, can be used as a marker of these early events [38,41]. However, there is a variable response to agonists that may also not necessarily be concentration-dependent. For example, intermediate doses of thrombin (0.5 nM) compared with higher doses (5 nM) were shown to induce higher levels of CD62P expression [38]. In taking Leytin’s index of platelet activation [42] further, our group similarly determined that CD41a+ platelets upregulate CD62P expression maximally at 0.1 U/mL, whereas at a higher concentration (0.25 U/mL), CD62P expression is rapidly lost [43]. This is indicative of platelet degranulation, which can be visualised using scanning electron microscopy, as membrane pores that extend into the open canalicular system can thus be regarded as a later period in the activation process [43].

Platelet shape changes were also identified to occur despite a significant reduction in degranulation in platelets deficient in the G-protein α subunit Gαq in response to thrombin, TXA_2_ and collagen [44]. Deficiency of the G-protein α subunits Gαi and Gα13 reduces the degranulation induced by ADP and thrombin, respectively [44]. This highlights the importance of unravelling intracellular signalling pathways in the penultimate responses to agonists and platelet–tumour cell-based interactions that may not result in standard behavioural prescripts. For example, tumour-induced platelet activation may induce the release of microparticles or microvesicles without causing platelet aggregation [45,46,47]. These microparticles not only express functional proteins but also contain bioactive molecules and miRNAs that can be transferred to neighbouring cells, including tumour cells [32,48]. Platelet activation can thus also stimulate the translation of multiple proteins and result in shed microparticles that act as a delivery system to regulate cell responses [32,49].

CD63 is a marker that is linked to a period of later platelet activation [50], whereby its expression on the platelet surface is predicated on its release from dense granules and lysosomes. It associates with the integrin αIIbβ3-CD9 complex and the actin cytoskeleton and is thus implicated in membrane spreading [51]. Our research group showed that even low levels of CD63 expression correlate with CD62P in healthy, whole blood [47]. Flow cytometry results are thus able to explain the distribution of platelet activation markers in response to agonists, highlighting a heterogeneous response to stimulants and that some platelets may indeed be unresponsive [38,47,52,53].

A refractory response to agonists was also suggested to reflect platelet exhaustion, whereby platelets that are in a higher state of activation and are unable to upregulate CD62P or CD63 expression and aggregate are nevertheless prone to increase the risk of thrombosis, as identified in cases of traumatic brain injury and viral haemorrhagic diseases [36,43,54]. Moreover, in cancer, the induction of a platelet secretory phenotype also increases the risks associated with tumour growth and spread. Localised inflammation mediated by the release of cytokines and chemokines is also associated with hypercoagulation through the release of coagulation factors from recruited monocytes and macrophages [55]. Modulating platelet capacity to respond to coagulation agonists was thus postulated to limit the reciprocal interactions that drive tumour progression and, consequently, thrombosis and may be a potential target for the development of novel therapeutic targets.

## 3. Platelets in the Tumour Microenvironment

The TME acts as a double-edged sword by facilitating tumour progression and hypercoagulation. In the TME, platelets interact with tumour cells within the tumour bed and the vasculature. Fibrin or platelet plugs in and around many types of tumours were observed by histological analyses, implying local activation of the coagulation process [56]. Extravasation of platelets into the tumour bed is dependent on proteins involved in adhesion, including CD62P and platelet focal adhesion kinase (FAK), and is permitted through dysfunctional tumour-associated vasculature [46]. These reciprocal interactions between tumour cells and platelets are postulated to enhance tumour survival and progression, with secondary consequences of inducing a hypercoagulatory environment.

Extravasated CD42b+ platelets were found in primary breast tumours, with definitive co-localisation with tumour cells at the invasive front and in the perivascular tissue [57]. These tumour cells showed positivity for markers of EMT, including loss of E-cadherin expression and heightened vimentin expression, with a loss of apicobasal polarity and detachment at the invasive front that is reflective of a more aggressive phenotype [57]. Similarly, CD42b+ platelets were identified at the invasive front of pancreatic ductal adenocarcinoma, with a significant correlation found with markers of EMT, increased Snail1 expression and decreased E-cadherin expression [58]. In gastric cancer, intratumoral platelets have been associated with poor prognosis [59] and chemoresistance [60]. In breast tumours, there were similar findings, but caution needs to be made regarding determining whether intratumoral platelet aggregation has a significant relationship with survival outcomes given the variance of pathological complete responses in the different breast tumour subtypes [57]. Chemotherapy is also able to enhance D-dimer presentation, which is a degradation product of fibrin and is thus used as a proxy of fibrin concentration [22]. Fibrinogen distribution in platelet α-granules is heterogeneous [61], as its release during platelet activation and the structural fibrin produced is dependent on the agonist and concentration thereof, with thrombin having a major role [62]. Fibrin network structure affects fibrinolysis, which when dysregulated, enhances the risk of VTE [63]. Platelet α-granules also contain the inhibitors of the coagulation cascade for homeostasis [61]. These inhibitors include anti-thrombin, protein C and protein S, and are markedly reduced in the plasma of cancer patients, thereby creating favourable conditions for hypercoagulation [56,64].

Platelet aggregation in the tumour bed has been associated not only with inducing and maintaining key EMT genes in tumour cells [65] but with a poor response to chemotherapy [57] and poor survival outcomes [66]. Preventing local platelet activation and the associated release of growth factors and cytokines may thus mitigate the induction of pro-tumorigenic processes and anti-apoptotic mechanisms that facilitate metastasis. Notably, platelet depletion studies in tumour-bearing murine models highlighted their fundamental role in the secretion of pro-angiogenic factors, including VEGF and TGF, and the development of tumour-associated vasculature [67,68,69]. These factors are additionally associated with the recruitment of myeloid-derived suppressor cells (MSDCs), which are also implicated in maintaining an immunosuppressive, tumour-permissive microenvironment [59].

CD97, which is a G-protein coupled receptor, involved in adhesion is upregulated on several solid tumours, whereby it facilitates trans-endothelial migration of tumour cells by inducing the release of platelet-derived lysophosphatidic acid that enhances tumour cell invasiveness and ATP that enhances vascular permeability [70]. Such bi-directional signalling underpins the relationship between tumour progression and the development of thrombosis. Tumour cells typically travel through the vascular system during invasion and metastasis and platelets form a shield or cloak around circulating tumour cells using molecules typically involved in adhesion, including GPIIb/IIIa, P-selectin, GPIb/V/IX and platelet-endothelial cell adhesion molecule-1 (PECAM1) [71]. This platelet cloak not only protects tumour cells from the high shear forces within the vasculature but also from immunosurveillance, for example, from NK cells [71]. Platelet-derived TGF-β1 induces the downregulation of NKG2D receptors, while the expression of glucocorticoid TNF-related ligand (GITRL) and the receptor of NF-κΒ ligand (RANKL) on the platelet membrane reduce NK cell cytolytic function [72,73,74]. Additionally, platelets and platelet-derived ectosomes can transfer MHC I molecules to tumour cells, leading to the inhibition of NK cell function [75,76]. Moreover, platelet–tumour cell conjugates, via the secretion of a host of cytokines, including the chemokines CXCL5, CXCL7 and RANTES, can recruit granulocytes and monocytes to prepare the pre-metastatic niche, enhance vascular permeability and facilitate the establishment of secondary sites [71].

Thrombin was also shown to not only be involved in the coagulation cascade but also promote tumour survival through PAR-1 and fibrinogen-mediated pathways [76]. Fibroblasts and macrophages within the TME secrete pro-tumorigenic agents, including monocyte chemotactic protein (MCP-1), following stimulation by thrombin [76]. Thrombin mediates angiogenesis by promoting the proliferation and differentiation of murine vascular progenitor cells [77] and the mitogenic effects of VEGF on endothelial cells to form leaky tumour-associated vessels adapted for transendothelial migration of tumour cells [76]. GPIIaIIIb expressed on both platelets and tumour cells has a bi-directional role in that it facilitates platelet adhesion and interactions with extracellular proteins and further facilitates the release of proangiogenic factors VEGF and bFGF in breast cancer and melanoma [76].

Potent angiogenic factors, including VEGF, PDGF, bFGF and EGF, and antiangiogenic factors (such as endostatin, angiostatin, PF4 and thrombospondin) are primarily stored in platelet alpha granule stores [76]. Tumour-associated endothelial cells have a high affinity for platelets through increased TF expression, which promotes thrombin production and the associated growth factor release [76]. Platelet microparticles were shown to increase the expression of VEGF in lung cancer [78]. CD154 (also known as CD40 ligand) expressed by platelets also promotes angiogenesis. Furthermore, angiogenesis is also achieved through the platelet-mediated recruitment of bone-marrow-derived progenitor cells to tumour tissue or hypoxic areas [76]. There are thus various pathways in which platelets promote angiogenesis within the TME to promote tumour cell survival.

Bi-directional signalling between breast tumour cells and platelets was also investigated in vitro, revealing that breast cancer cell lines, including MCF-7, MDA-MB-231 and T47D, are able to induce platelet activation and aggregation and that platelets, in turn, are able to induce more aggressive tumour phenotypes, promoting EMT and metastasis [45,47,79,80,81]. Clinically, treatment for hormone-dependent breast cancer, including hormone therapy, chemotherapy and surgery, have all been implicated in enhancing thromboembolic events [16]. While investigating this phenomenon in vitro, some studies identified that the selective oestrogen receptor modulator Tamoxifen is able to prevent platelet activation [82,83]; furthermore, our own in vitro assessments of Tamoxifen and the aromatase inhibitor Anastrozole rather echoed clinical studies that showed heightening of platelet reactivity and thrombotic propensity [45,52,84,85]. Such contradictions require further investigations to unravel variances in methodologies; however, the overall data point to the use of anti-platelet strategies as a mechanism to not only prevent thromboembolic complications but also to prevent platelet-mediated tumour progression.

## 4. Anti-Platelet Therapy in Thromboembolic Disease and Cancer

### 4.1. Platelet Activation—The Impact of Tumour Cells

Platelet activation is a precursor for thrombosis and can be mediated through various mechanisms in the vasculature and within the tumour microenvironment. Various platelet ligands, including TXA_2_, ADP and thrombin, bind to P2Y1 and P2Y12 receptors on platelets (Figure 1), and thus, activate them [86]. Platelets are also activated through tyrosine binding to immunoreceptors on their surface, including c-type lectin-like receptor-2 (CLEC-2), glycoprotein VI (GPVI) and FcRγIIA receptor [76,87]. Tumour cells release microparticles that contain ligands for P-selectin on platelets, including TF and P-selectin glycoprotein ligand-1 (PSGL-1) [87]. Such reciprocal interactions can also be mediated by structural components of the coagulation cascade, whereby activated platelets bind to fibrinogen on tumour cells through GPIIbIIIa. This enhances platelet activation through the RapIb-GTP and phospholipase C pathways [87]. Tumour cells release high-mobility group box 1, which binds to toll-like receptor 4 (TLR4) on platelets and leads to their activation [76]. These direct interactions and others between tumour cells and platelets are termed tumour-cell-induced platelet activation (TCIPA) and typically occur upon the entry of tumour cells into circulation, whereby platelets bind to them, forming a protective shield [87].

TCIPA is characterised by the following types of aggregations: platelet–platelet, platelet–tumour and tumour–platelet–leukocytes [87]. In vitro studies that mimicked TCIPA were variable and impacted, for example, by centrifugation speed, the use of adherent tumour cells or those in suspension, and wash steps [43,79,84]. Additionally, the fact that the processes of platelet activation and aggregation while incorporating similar molecular events are fundamentally different makes it difficult to adequately recapitulate in vivo events in vitro. Nevertheless, research must strive to untangle these important interactions. In vivo, TCIPA provides protection from high shear stress and facilitates immune evasion within the bloodstream [87]. TCIPA is determined by the metastatic potential of the tumour, and thus, would vary across different tumour types [87]. Metastatic potential and invasive capacity are regulated to a large degree by the TGF-β/Smad and NF-kβ signalling pathways. These pathways can be activated by platelet-derived TGF-β and direct contact with integrin α2β1 [87]. The release of platelet-derived TGF-β can also be induced by interactions between CLEC-2 and podoplanin on tumour cells [87]. Tumour cells were shown to directly and indirectly affect the transcriptome profile of platelets, thus leading to tumour-educated platelets (TEP) through the transfer of RNA or regulating platelet mRNA, miRNA, circRNA, IncRNA and mitRNA through the PMP transfer of regulatory mRNA into other cell types within the TME [87]. TEP are novel biomarkers for cancer and could be potential therapeutic targets.

The role of antiplatelet therapies on the incidence of cancer is reviewed here by presenting three major pathways that are involved in platelet activation during thrombosis, namely, cyclooxygenase (COX) function in TXA_2_ synthesis, engagement of the P2Y12 receptors by ADP and engagement of the protease-activated receptor (PAR1) by thrombin [88,89]. While this is not an exhaustive list, targeting these pathways has shown considerable efficacy in reducing thrombotic events related to cardiovascular disease; however, there remains much research that is required on the impact of such drugs on cancer incidence and progression.

### 4.2. Inhibition of Cyclooxygenase (COX) Enzymes with Aspirin

Aspirin (acetylsalicylic acid), along with other classical non-steroidal anti-inflammatory drugs (NSAIDs), competes with arachidonic acid to bind irreversibly to COX enzymes [90,91], thereby blocking the biosynthesis of cyclic prostanoids, including TXA_2_ and other prostaglandins [92,93,94]. There are two isoforms of the COX enzyme, namely, COX-1 (classical) and COX-2 (inducible), with the former primarily associated with platelets and the epithelium of the gastric mucosa. COX-2 has low to no expression in platelets [95], but is overexpressed in tumours of the pancreas, lung and breast [96]. Under physiological conditions, TXA_2_ binds to TP receptors, causing platelet activation and aggregation and facilitating thrombosis [88,97]. Even low-dose Aspirin is able to prevent these intrinsic platelet processes despite Aspirin’s short half-life (20 min) [96]. Similarly, prostaglandins, which are local mediators of normal cellular reactions, have a short lifespan, although they increase during inflammation and in tumours [98]. Prostaglandin H2 (PgH2), the precursor of all prostaglandins, is induced in response to elevated COX expression in breast tissue, stimulating the activity of the aromatase enzyme CYP19, and thus, increasing oestrogen synthesis [99]. High levels of COX-2, PgH2 and prostaglandin E2 (PGE2) are associated with metastatic potential and reduced patient survival, reflecting oestrogen-mediated downstream cellular functions, including growth, proliferation and migration [99,100,101]. COX-2 has thus been positioned as a key factor in enhancing oestrogen production and treatment failure in hormone-dependent breast tumours [102]. Studies thus suggested that Aspirin could be useful as an alternative treatment or to enhance known therapies for hormone-dependent breast cancer, which is indirectly linked to the suppression of aromatase activity, and thus, a reduction in oestrogen production [102,103,104].

In a murine model, the effect of Aspirin on cancer was shown to be dose-dependent, with anti-metastatic effects induced by low-to-medium dosages of Aspirin inhibiting TXA_2_ production; however, the inhibition of COX2/PGE2 was not associated with anti-metastatic effects [105]. TXA_2_ generated through the COX-1-dependant pathway is associated with metastasis, hence Aspirin usage is shown to reduce metastasis. TXA2 signalling was also shown to activate the endothelium, promoting tumour cell survival by mediating the recruitment of pro-metastatic monocytes in proximity to tumour cells through the release of the chemotactic factors CCL2/MCP-1 and CCL5 [105]. Overall, COX-1 and TXA_2_ have a great impact on the development of the intravascular metastatic niche. While Aspirin effectively reduces these effects, it adversely affects the gastrointestinal tract with prolonged usage. As such, other selective TXA_2_ inhibitors, such as Picotamide, may be used as an alternative treatment, as it preserves the production of gastroprotective PGI2 [105].

Several studies pointed to an association between low-dose Aspirin use and reduced breast cancer risk and breast-cancer-related mortality; however, the results are inconsistent, with some studies indicating no association [106,107,108]. Yet other studies indicated that Aspirin use after diagnosis, rather than prophylactic use, is associated with improved survival outcomes of breast cancer patients [109]. A recent meta-analysis highlighted that while Aspirin use was linked with improved survival outcomes and reduced recurrence and metastasis, the dose and duration of Aspirin treatment were possible influencing factors that could account for the variation in numerous studies [110]. In contrast, the Aspirin Reduces Risk of Initial Vascular Events (ARRIVE) trial showed that cancer incidence increased with the routine use of Aspirin [87]. The Aspirin in Reducing Events in the Elderly (ASPREE) trial showed increased rates of gastrointestinal bleeding with prolonged use of low-dose Aspirin in healthy elderly adults and no reduction of tumorigenesis was observed; however, the findings of this study were limited by the short follow-up period (less than 5 years) and the advanced age of participants, which pre-disposed them to tumours [111]. The pharmacodynamics of aspirin may thus hold the key to understanding its role in tumour progression.

To achieve the inhibition of COX signalling in nucleated tumour cells, which, unlike platelets, have the capacity to synthesise de novo COX-1/2 enzymes, much higher and more frequent doses of Aspirin need to be administered [23,96]. However, caution is indicated since the inhibition of COX-1 may cause gastrointestinal toxicity, while the inhibition of COX-2 has been associated with an increased risk of cardiovascular and thrombotic events [112]. While Aspirin preferentially inhibits COX-1 to a larger degree than COX-2, pre-clinical models highlight that Aspirin inhibition of TXA_2_ synthesis, which is also produced directly by tumour cells [41,112], may underlie its key role in inhibiting breast tumour progression. The knockdown of TBXAS1 (the rate-limiting enzyme involved in TXA_2_ biosynthesis) and TBXA2R (TXA_2_ receptor) both of which are overexpressed in breast tumours, reduced the colony formation and proliferation of hormone-dependent breast tumour cell lines [112]. While aspirin acts to suppress TXA_2_-dependent platelet activation and aggregation, platelets may still retain their responsiveness to other agonists in healthy individuals [113]. For this reason, that Aspirin is likely unable to inhibit TCIPA [79,114]. In vitro assays have also determined that inhibiting platelet activation using Aspirin can prevent IL-8-driven invasion via engagement of the Akt pathway [115], highlighting other indirect avenues by which Aspirin may mediate tumour progression.

### 4.3. Blocking of ADP Receptors: Clopidogrel

Thienopyridines are a family of antiplatelet agents that bind irreversibly to platelet purinergic receptors and consist of three clinically approved drugs, namely, Ticlopidine, Clopidogrel and Prasugel [116,117], and nonthienopyridines, including Elinogrel, Ticagrelor and Cangrelor [118]. ADP is an agonist of platelet activation and elicits its action by binding to P2Y12 receptors, and thus, inhibiting the formation of cyclic adenosine monophosphate (cAMP) [119,120]. Various adenine nucleotides and nucleosides, i.e., adenosine triphosphate (ATP) and ADP, play a role in platelet aggregation and activation [121]. ATP is catalysed by ecto-nucleoside triphosphate diphospho-hydrolase (CD39) and ecto-5′-nucleotidase (CD73) into ADP, which is then converted into adenosine monophosphate (AMP); ultimately, AMP is converted into adenosine [121]. Tumour-derived ADP activates platelets via binding to P2Y12 receptors on the surface of platelets. Upon ADP binding, platelet aggregation is induced through the phospholipase-C- and phosphatidlyinositol-signalling pathways [119]. In vitro, ADP was highlighted as a secondary agonist that is essential to TCIPA, with a critical role for the P2Y12 receptor [79]. The efficacy of Clopidogrel against cardiovascular disorders is well established; however, novel agents, such as Prasugrel and Ticagrelor, were shown to be more potent and display a quicker onset of action than Clopidogrel [118].

Clopidogrel is a prodrug that is metabolised in the liver and converted to its active metabolite whereby it blocks the ADP P2Y12 platelet receptor in an irreversible manner [122,123]. Cytochrome P450 enzymes are involved in the metabolism of Clopidogrel, converting Clopidogrel into 2-oxo-Clopidogrel and subsequently into its active metabolite, as shown by in vitro studies [124,125]. CYP2C19 is the major enzyme involved in both oxidation steps in the conversion of Clopidogrel, whereas other enzymes, such as CYP34A, are only involved in one step. The active metabolite of Clopidogrel binds to a free cysteine on the P2Y12 receptor via its thiol group (only expressed in its active state). This binding irreversibly blocks the binding of ADP and subsequent activation of the purinergic receptor [124,125].

Clopidogrel is the most commonly used P2Y12 antagonist and is an effective agent against cardiovascular disorders, including peripheral artery cerebrovascular and coronary artery diseases [126]. While beneficial, its prolonged use is associated with an increased risk for bleeding and it was also shown to increase the mortality rate in patients with cardiovascular disease [127]. The dual administration of antiplatelet therapy with Aspirin and Clopidogrel is often recommended in certain conditions, such as coronary syndromes and myocardial infarction [128,129,130], because their efficacy is better when used as a cocktail than individually [88,117]. Studies showed that such dual treatment is beneficial in preventing the reoccurrence of atherothrombosis [128,129,130]; however, the effect on cancer is not well known. The Prevention of Cardiovascular Events in Patients with Prior Heart Attack Using Ticagrelor Compared to Placebo on a Background of Aspirin–Thrombolysis in Myocardial Infarction 54 (PEGASUS-TIMI 54) trial reported that prolonged use of Clopidogrel and Ticagrelor increased cancer-related deaths [131]. In the Clopidogrel versus Aspirin in Patients at Risk of Ischaemic Events (CAPRIE) study, combined Aspirin and Clopidogrel reduced the risk of myocardial infarction, stroke and vascular death [117]. The Clopidogrel for The Reduction of Events During Observation (CREDO) and Clopidogrel and Metoprolol Myocardial Infarction Trial (COMMIT) clinical trials showed that cardiovascular deaths were reduced with the use of Clopidogrel [117]. The efficacy of Clopidogrel was also shown in the Clopidogrel in Unstable Angina to Prevent Recurrent Events Trial (CURE) and Dual Antiplatelet Therapy (DAPT) clinical trials, whereby prolonged use decreased the incidence of myocardial infarction (MI) [127,132,133]. The Atrial Fibrillation Clopidogrel Trial with Irbesartan for Prevention of Vascular Events (ACTIVE-A) clinical trial showed a reduced incidence of stroke [127,132,133].

Breast tumours have the capacity to metabolise Clopidogrel into its active components via the expression of CYP450 family members. In vitro, breast cancer cells were shown to modulate the expression of purinergic receptors, suggesting a possible protective effect of this receptor against cancer [119]. Studies showed various outcomes regarding the use of Clopidogrel with or without Aspirin and cancer risk. The Clopidogrel for High Atherothrombotic Risk and Ischemic Stabilization, Management and Avoidance (CHARISMA) clinical trial showed that patients treated with a combination of Aspirin and Clopidogrel demonstrated a 54% decreased cancer incidence compared with Aspirin use alone, which reduced cancer incidence by 46% [134]. The reduction in cancer incidence with combined Aspirin and Clopidogrel was maintained for a prolonged period in solid tumours; however, no reduction was observed in hematologic tumours [134]. Other clinical trials, such as the Secondary Prevention of Small Subcortical Stroke (SPS3) and DAPT trials, showed that prolonged use of Clopidogrel was associated with an increased risk of cancer-related death [127,135]. The Trial to Assess Improvement in Therapeutic Outcomes by Optimizing Platelet Inhibition with Prasugrel Thrombolysis in Myocardial Infarction 38 (TRITON-TIMI 38) clinical trial showed that Prasugrel led to a higher risk for breast, colorectal and prostate cancer in patients with an acute coronary syndrome than Clopidogrel [136]. Clinical data is thus suggestive of adverse effects, the mechanisms of which need to be investigated further, starting at a pre-clinical level.

Mouse models have highlighted that an Aspirin and Clopidogrel cocktail induced pro-tumorigenic alterations in primary tumours, increased angiogenesis through vascular mimicry and was associated with high mortality rates [137]. Similarly, Clopidogrel combined with Tamoxifen and chemotherapy agents (Cisplatin, Doxorubicin) increased metastasis to the murine lung [120]. In vitro assessments showed that an Aspirin and Clopidogrel cocktail failed to reduce the hypercoagulatory effects induced by Anastrozole in breast cancer cell lines; moreover, there was evidence for a tumour cell EMT characterised by the upregulation of N-cadherin and production of the cytokines IL-6, PDGF-BB and TGF-β3 [47]. Notably, T47D cells, which are from a more aggressive luminal-A breast cancer cell line, assumed a more invasive phenotype under an Anastrozole, Aspirin and Clopidogrel cocktail, with morphological features related to enhanced survival and motility [138].

### 4.4. Targeting the Thrombin Pathway with Atopaxar

Thrombin is the main extracellular serine protease responsible for activating PAR1, PAR3 and PAR4 receptors [139,140]. PAR1 is the main receptor for thrombin binding in platelets, and PAR receptors (predominately PAR1 and PAR2) are widely expressed in tumour cells and were shown to mediate pro-survival processes, such as tumour growth, cell proliferation, migration, invasion, inflammation and angiogenesis [140]. PAR3 and PAR4 receptors were discovered as novel thrombin receptors when PAR1 was knocked out in a murine model [141]. PAR4 is activated by higher levels of thrombin than PAR1 due to a lack of a hirudin-like domain. PAR1 and PAR3 both contain the hirudin-binding site, which offers high-affinity binding for thrombin [141]. In contrast, PAR2 has a very low affinity for thrombin; however, at higher thrombin concentrations (100–500 nM), it may be susceptible to thrombin activation [142].

Thrombin is a potent agonist for platelet activation and is an essential component of the coagulation cascade. Thrombin activates PAR1 receptors in two stages: first by binding to the cleavage site on either side of the PAR1 receptors, then cleaving the PAR1 receptor between Arg and Ser, exposing the N-terminal tethered ligand domain SFLLRN [143,144,145]. The tethered ligand domain interacts with domains in extracellular loop2, which was suggested to alter the conformation of the receptors to allow for the coupling of G-proteins [143,145]. Other proteases that cleave PAR receptors include anticoagulant protease-activated protein C (aPC), which co-localises with the endothelial protein C receptor (EPCR) to activate PAR1 and also acts on PAR3 in humans [146]. Coagulation factor Xa also activates PAR1, PAR2 and PAR3 when bound to EPCR. Coagulation factor VIIa bound to TF indirectly activates PAR2 through the cleavage of matriptase [146].

Cancer patients often present with elevated levels of thrombin, and this is associated with a poor prognosis [147]. In the Vienna Cancer and Thrombosis Study (CATS), which included 1033 cancer patients, thrombin levels were assessed using the calibrated automated thrombogram (CAT) method and increased levels of thrombin were associated with VTE occurrence in 77 patients (~7%) after 2 years [148]. The TME is a hotspot for thrombin production, with breast cancer cells capable of directly producing thrombin to initiate platelet activation and aggregation [45,79,147]. Moreover, thrombin also facilitates fibrin formation that supports platelet aggregation via GPIIbIIIa [79] and subsequent inside-out signalling. Notably, in vitro, tumour-induced fibrin network formation is also variable and may hint at impaired fibrinolytic mechanisms. While there have been some assertions that in vitro TCIPA may not be as dependent on tumour phenotype [79], other in vitro studies indicated that not only does the tumour sub-phenotype matter but different sub-phenotypes in the case of breast cancers also respond differentially to hormone therapy, which, in turn, affects their capacity to induce hypercoagulation [52,84,138]. This may also be reflected clinically by the range of thrombotic risk associated with various types of tumours [2,16,17,149].

PAR-1 receptor expression in various cancer types, including breast, lung and ovarian cancer, is associated with a poor prognosis [150]. PAR1 inhibitors, including Atopaxar, Vorapaxar and PZ-128, which have reciprocal effects in reducing platelet aggregation and through direct interaction with PAR1 receptors on tumour cells, were shown to reduce metastasis [151]. The downstream effects of PAR1 inhibition were shown to be dependent on the tumour type. The inhibition of PAR1 through gene silencing in a breast cancer xenograft model caused reduced metastasis to the lung; however, this was possibly a limitation of using an immunocompromised animal model, which could not account for possible immune influence on tumour progression [151]. However, the treatment of breast cancer cell lines with Vorapaxar inhibited migration and invasion in vitro [151], while in pancreatic cancer, PAR1 signalling was shown to mediate hypercoagulation and immune escape [152]; therefore, PAR-1 inhibitors could play an important therapeutic role in this tumour by mediating different aspects of tumour progression.

Atopaxar and Vorapaxar are small non-peptide molecules that interact with PARs through ECL2, and thus, inhibit receptor binding [141]. There are few targets that have been identified and developed as potential therapeutic targets against PAR-mediated activities, despite the role of PARs in mediating the cellular processes that underlie disease processes [141]. Vorapaxar and Atopaxar were the first thrombin receptor antagonists developed that selectively block the platelet PAR1 receptor subtypes [143,145,153]. Vorapaxar was approved by the FDA for clinical use in cardiovascular patients despite its adverse effect of increased bleeding incidence [154,155]. Most other PAR antagonists are still in the early stages of clinical development for their efficacy against cardiovascular diseases [155]. The Lessons for Antagonizing the Cellular Effect of Thrombin-Acute Coronary Syndrome (LANCELOT-CAS) clinical trial showed Atopaxar’s efficacy in reducing ischemic events and platelet aggregation; unlike most antiplatelet agents (such as Aspirin, Clopidogrel and Vorapaxar), it did not adversely increase the bleeding time [156]. Furthermore, Atopaxar was shown to inhibit thrombin receptor activating peptide (TRAP) [154]; however, together with The Japanese Lessons from Antagonizing the Cellular Effects of Thrombin (J-LANCELOT) clinical trial, Atopaxar was found to adversely affect liver function, and thus, phase III clinical studies were not conducted [157]. The role of PAR receptor antagonists on direct tumour outcome has not been well established, and given the impact of PAR-receptor mediated signalling in promoting tumour survival [23,158], the role of PAR receptor antagonists, such as Atopaxar, in preventing PAR signalling presents a potential adjuvant therapy that warrants further investigation.

Atopaxar is metabolised by the cytochrome P450 enzyme CYP3A4 [128]. Breast tumour cell lines express targets for Atopaxar and metabolizing enzymes [159], with higher PAR-1 expression associated with heightened invasiveness and induction of EMT [160]. Tumour cell production of thrombin in vivo and in vitro facilitates PAR signalling and downstream effects with the TME [79,138]. A recent in vitro study showed that Atopaxar is an antagonist to the JAK-STAT signalling pathway, which promotes cell division and neoplastic transformation in cells, thus suggesting a role for Atopaxar in reducing tumour formation [161]. In our lab, in vitro tests showed that Atopaxar failed to prevent hypercoagulation induced by breast cancer cell lines, with platelets showing evidence of heightened activation and aggregation. This was coupled with the induction of a partial EMT, particularly in MCF7 cells, that was underwritten by IL-6, TGF-α and TGF-β3 secretion [47,138]. These findings show that Atopaxar failed to mitigate tumour-induced hypercoagulation or to reduce features that mediate tumour survival in vitro; however, further studies are required in order to elucidate its role in tumour-promoting or -inhibiting processes given such conflicting findings.

## 5. Conclusions

Bi-directional signalling between platelets and tumour cells creates favourable circumstances for tumour progression, with thrombotic complications being necessary side effects by virtue of the associations between the cell types. While more is known about the role of Aspirin in preventing metastasis, less is known regarding the prevention of early tumour processes and what effects drugs targeting other activation pathways, including Clopidogrel and Atopaxar, may have. What is becoming clearer is the complexity of platelet activation and the plethora of growth factors, cytokines and coagulation agonists that may be released in response to indirect or direct tumour signals. That these factors impact tumour progression is increasingly evident, with the need to prevent platelet activation, if not aggregation, also evident. However, the drugs used, as well as their combination with standard cancer therapeutics, may not yield the desired beneficial effects against thromboembolic disease but may act as a selective pressure for tumour survival. It is increasingly evident that further research to recapitulate the clinical tumour microenvironment more faithfully in vitro and in in vivo animal models is necessary to provide systems in which to unpack these complex interactions.

## Data Availability

Data sharing not applicable.

## References

[1] Martin K.A., Molsberry R., Khan S.S., Linder J.A., Cameron K.A., Benson A. (2020). Preventing Venous Thromboembolism in Oncology Practice: Use of Risk Assessment and Anticoagulation Prophylaxis. Res. Pract. Thromb. Haemost..

[2] Donnellan E., Khorana A.A. (2017). Cancer and Venous Thromboembolic Disease: A Review. Oncologist.

[3] Menter D.G., Tucker S.C., Kopetz S., Sood A.K., Crissman J.D., Kenneth V. (2014). Platelets and Cancer: A Casual or Causal Relationship: Revisited. Cancer Metastasis Rev..

[4] Sung H., Ferlay J., Siegel R.L., Laversanne M., Soerjomataram I., Jemal A., Bray F. (2021). Global Cancer Statistics 2020: GLOBOCAN Estimates of Incidence and Mortality Worldwide for 36 Cancers in 185 Countries. CA Cancer J. Clin..

[5] Sharma R., Aashima, Nanda M., Fronterre C., Sewagudde P., Ssentongo A.E., Yenney K., Arhin N.D., Oh J., Amponsah-Manu F. (2022). Mapping Cancer in Africa: A Comprehensive and Comparable Characterization of 34 Cancer Types Using Estimates from GLOBOCAN 2020. Front. Public Health.

[6] Siegel R.L., Miller K.D., Fuchs H.E., Jemal A. (2022). Cancer Statistics, 2022. CA Cancer J. Clin..

[7] Kochel T.J., Reader J.C., Ma X., Kundu N., Fulton A.M. (2017). Multiple Drug Resistance-Associated Protein (MRP4) Exports Prostaglandin E2 (PGE2) and Contributes to Metastasis in Basal/Triple Negative Breast Cancer. Oncotarget.

[8] De Lima Mota A., Evangelista A.F., Macedo T., Oliveira R., Scapulatempo-Neto C., Vieira R.A., Marques M.M.C. (2017). Molecular Characterization of Breast Cancer Cell Lines by Clinical Immunohistochemical Markers. Oncol. Lett..

[9] Polyak K. (2011). Heterogeneity in Breast Cancer. J. Clin. Investig..

[10] Martelotto L.G., Ng C.K.Y., Piscuoglio S., Weigelt B., Reis-Filho J.S. (2014). Breast Cancer Intra-Tumor Heterogeneity. Breast Cancer Res..

[11] Eliyatkin N., Yalcin E., Zengel B., Aktaş S., Vardar E. (2015). Molecular Classification of Breast Carcinoma: From Traditional, Old-Fashioned Way to A New Age, and A New Way. J. Breast Health.

[12] Rotimi S.O., Rotimi O.A., Salhia B. (2021). A Review of Cancer Genetics and Genomics Studies in Africa. Front. Oncol..

[13] Vitale I., Shema E., Loi S., Galluzzi L. (2021). Intratumoral Heterogeneity in Cancer Progression and Response to Immunotherapy. Nat. Med..

[14] Brothwell M.R.S., Barnett G.C. (2022). Cancer Genetics and Genomics e Part 2 Somatic Genetic Changes in Cancer Cells. Clin. Oncol..

[15] Zhang Q., Liu H., Zhu Q., Zhan P., Zhu S., Zhang J., Lv T., Song Y. (2017). Patterns and Functional Implications of Platelets upon Tumor “Education”. Int. J. Biochem. Cell Biol..

[16] Razak N.B.A., Jones G., Bhandari M., Berndt M.C., Metharom P. (2018). Cancer-Associated Thrombosis: An Overview of Mechanisms, Risk Factors, and Treatment. Cancers.

[17] Papaxoinis G., Kamposioras K., Germetaki T., Weaver J.M.J., Stamatopoulou S., Nasralla M., Kordatou Z., Owen-Holt V., Anthoney A., Mansoor W. (2018). Predictive Factors of Thromboembolic Complications in Patients with Esophagogatric Adenocarcinoma Undergoing Preoperative Chemotherapy. Acta Oncol..

[18] Koupenova M., Kehrel B.E., Corkrey H.A., Freedman J.E. (2017). Thrombosis and Platelets: An Update. Eur. Heart J..

[19] Holinstat M., Yeung J. (2012). Newer Agents in Antiplatelet Therapy: A Review. J. Blood Med..

[20] Key N.S., Khorana A.A., Mackman N., McCarty O.J.T., White G.C., Francis C.W., McCrae K.R., Palumbo J.S., Raskob G.E., Chan A.T. (2016). Thrombosis in Cancer: Research Priorities Identified by a National Cancer Institute/National Heart, Lung, and Blood Institute Strategic Working Group. Cancer Res..

[21] Fennerty A. (2006). Venous Thromboembolic Disease and Cancer. Postgrad. Med. J..

[22] Wang Z.H.I., Dang C., Zhu K.U.N., Zhang Y., Chang D., Xia P., Song Y., Li K. (2015). Cyclophosphamide, Epirubicin and Fluorouracil Chemotherapy-Induced Alteration of Haemostasis Markers in Breast Cancer Patients. Mol. Clin. Oncol..

[23] Haemmerle M., Stone R.L., Menter D.G., Afshar-Kharghan V., Sood A.K. (2018). The Platelet Lifeline to Cancer: Challenges and Opportunities. Cancer Cell.

[24] Lin R.J., Afshar-Kharghan V., Schafer A.I. (2014). Paraneoplastic Thrombocytosis: The Secrets of Tumor Self-Promotion. Blood.

[25] Bailey S.E.R., Ukoumunne O.C., Shephard E., Hamilton W. (2017). How Useful Is Thrombocytosis in Predicting an Underlying Cancer in Primary Care? A Systematic Review. Fam. Pract..

[26] Voutsadakis I.A. (2014). Thrombocytosis as a Prognostic Marker in Gastrointestinal Cancers. World J. Gastrointest. Oncol..

[27] Golebiewska E.M., Poole A.W. (2015). Platelet Secretion: From Haemostasis to Wound Healing and Beyond. Blood Rev..

[28] Clemetson K.J. (2012). Platelets and Primary Haemostasis. Thromb. Res..

[29] Weber M.R., Zuka M., Lorger M., Tschan M., Torbett B.E., Zijlstra A., Quigley J.P., Staflin K., Eliceiri B.P., Krueger J.S. (2016). Activated Tumor Cell Integrin Avβ3 Cooperates with Platelets to Promote Extravasation and Metastasis from the Blood Stream. Thromb. Res..

[30] Zhang N., Newman P.J. (2019). Packaging Functionally Important Plasma Proteins into the α-Granules of Human-Induced Pluripotent Stem Cell-Derived Megakaryocytes. J. Tissue Eng. Regen. Med..

[31] Leblanc R., Lee S.C., David M., Bordet J.C., Norman D.D., Patil R., Miller D., Sahay D., Ribeiro J., Clézardin P. (2014). Interaction of Platelet-Derived Autotaxin with Tumor Integrin AVb3 Controls Metastasis of Breast Cancer Cells to Bone. Blood.

[32] Lazar S., Goldfinger L.E. (2018). Platelet Microparticles and MiRNA Transfer in Cancer Progression: Many Targets, Modes of Action, and Effects Across Cancer Stages. Front. Cardiovasc. Med..

[33] Van Holten T.C., Bleijerveld O.B., Wijten P., De Groot P.G., Heck A.J.R., Barendrecht A.D., Merkx T.H., Scholten A., Roest M. (2014). Quantitative Proteomics Analysis Reveals Similar Release Profiles Following Specific PAR-1 or PAR-4 Stimulation of Platelets. Cardiovasc. Res..

[34] Pokrovskaya I.D., Aronova M.A., Kamykowski J.A., Prince A.A., Hoyne J.D., Calco G.N., Kuo B.C., He Q., Leapman R.D., Storrie B. (2016). STEM Tomography Reveals That the Canalicular System and α-Granules Remain Separate Compartments during Early Secretion Stages in Blood Platelets. J. Thromb. Haemost..

[35] Peters C.G., Michelson A.D., Flaumenhaft R. (2012). Granule Exocytosis Is Required for Platelet Spreading: Differential Sorting of α-Granules Expressing VAMP-7. Blood.

[36] Baaten C.C.F.M.J., ten Cate H., van der Meijden P.E.J., Heemskerk J.W.M. (2017). Platelet Populations and Priming in Hematological Diseases. Blood Rev..

[37] Kawato M., Shirakawa R., Kondo H., Higashi T., Ikeda T., Okawa K., Fukai S., Nureki O., Kita T., Horiuchi H. (2008). Regulation of Platelet Dense Granule Secretion by the Ral GTPase-Exocyst Pathway. J. Biol. Chem..

[38] Ollivier V., Syvannarath V., Gros A., Butt A., Loyau S., Jandrot-Perrus M., Ho-Tin-Noé B. (2014). Collagen Can Selectively Trigger a Platelet Secretory Phenotype via Glycoprotein VI. PLoS ONE.

[39] Van Asten I., Schutgens R.E.G., Baaij M., Zandstra J., Roest M., Pasterkamp G., Huisman A., Korporaal S.J.A., Urbanus R.T. (2018). Validation of Flow Cytometric Analysis of Platelet Function in Patients with a Suspected Platelet Function Defect. J. Thromb. Haemost..

[40] Ramström S., Södergren A.L., Tynngård N., Lindahl T.L. (2016). Platelet Function Determined by Flow Cytometry: New Perspectives?. Semin. Thromb. Hemost..

[41] Schwarz S., Gockel L.M., Naggi A., Barash U., Gobec M., Bendas G., Schlesinger M. (2020). Glycosaminoglycans as Tools to Decipher the Platelet Tumor Cell Interaction: A Focus on P-Selectin. Molecules.

[42] Leytin V., Mody M., Semple J.W., Garvey B., Freedman J. (2000). Quantification of Platelet Activation Status by Analyzing P-Selectin Expression. Biochem. Biophys. Res. Commun..

[43] Augustine T.N., Van Der Spuy W.J., Kaberry L.L., Shayi M. (2016). Thrombin-Mediated Platelet Activation of Lysed Whole Blood and Platelet-Rich Plasma: A Comparison between Platelet Activation Markers and Ultrastructural Alterations. Microsc. Microanal..

[44] Cho M.S., Li J., Gonzalez-Delgado R., Lee H., Vasquez M., He T., He Y., Liu K., Sasano T., Nürnberg B. (2021). The Effect of Platelet G Proteins on Platelet Extravasation and Tumor Growth in the Murine Model of Ovarian Cancer. Blood Adv..

[45] Pather K., Augustine T.N. (2020). Tamoxifen Induces Hypercoagulation and Alterations in ERα and ERβ Dependent on Breast Cancer Sub-Phenotype Ex Vivo. Sci. Rep..

[46] Plantureux L., Mege D., Crescence L., Carminita E., Robert S., Cointe S., Brouilly N., Ezzedine W., Dignat-George F., Dubois C. (2020). The Interaction of Platelets with Colorectal Cancer Cells Inhibits Tumor Growth but Promotes Metastasis. Cancer Res..

[47] Xulu K.R., Augustine T.N. (2021). Antiplatelet Therapy Combined with Anastrozole Induces Features of Partial EMT in Breast Cancer Cells and Fails to Mitigate Breast–Cancer Induced Hypercoagulation. Int. J. Mol. Sci..

[48] Burnouf T., Goubran H.A., Chou M.L., Devos D., Radosevic M. (2014). Platelet Microparticles: Detection and Assessment of Their Paradoxical Functional Roles in Disease and Regenerative Medicine. Blood Rev..

[49] Meikle C.K.S., Kelly C.A., Garg P., Wuescher L.M., Ali R.A., Worth R.G. (2017). Cancer and Thrombosis: The Platelet Perspective. Front. Cell Dev. Biol..

[50] Heemskerk J.W.M., Mattheij N.J.A., Cosemans J.M.E.M. (2013). Platelet-Based Coagulation: Different Populations, Different Functions. J. Thromb. Haemost..

[51] Blair T.A., Michelson A.D., Frelinger A.L. (2018). Mass Cytometry Reveals Distinct Platelet Subtypes in Healthy Subjects and Novel Alterations in Surface Glycoproteins in Glanzmann Thrombasthenia. Sci. Rep..

[52] Pather K., Dix-Peek T., Duarte R., Chetty N., Augustine T.N. (2019). Breast Cancer Cell-Induced Platelet Activation Is Compounded by Tamoxifen and Anastrozole in Vitro. Thromb. Res..

[53] Eckly A., Rinckel J.Y., Proamer F., Ulas N., Joshi S., Whiteheart S.W., Gachet C. (2016). Respective Contributions of Single and Compound Granule Fusion to Secretion by Activated Platelets. Blood.

[54] Riedl J., Riedl J., Pabinger I., Ay C. (2014). Platelets in Cancer and Thrombosis. Hamostaseologie.

[55] Koltsova E.M., Sorokina M.A., Pisaryuk A.S., Povalyaev N.M., Ignatova A.A., Polokhov D.M., Kotova E.O., Balatskiy A.V., Ataullakhanov F.I., Panteleev M.A. (2021). Hypercoagulation Detected by Routine and Global Laboratory Hemostasis Assays in Patients with Infective Endocarditis. PLoS ONE.

[56] Prandoni P., Falanga A., Piccioli A. (2006). Cancer and Venous Thromboembolism. Clinical Implications of Strong Association. Pathophysology Haemost. Thromb..

[57] Ishikawa S., Miyashita T., Inokuchi M., Hayashi H., Oyama K., Tajima H., Takamura H., Ninomiya I., Ahmed A.K., Harman J.W. (2016). Platelets Surrounding Primary Tumor Cells Are Related to Chemoresistance. Oncol. Rep..

[58] Miyashita T., Tajima H., Makino I., Nakagawara H., Kitagawa H., Fushida S., Harmon J.W., Ohta T. (2015). Metastasis-Promoting Role of Extravasated Platelet Activation in Tumor. J. Surg. Res..

[59] Yamaguchi T., Fushida S., Kinoshita J., Okazaki M., Ishikawa S., Ohbatake Y., Terai S., Okamoto K., Nakanuma S., Makino I. (2020). Extravasated Platelet Aggregation Contributes to Tumor Progression via the Accumulation of Myeloid-Derived Suppressor Cells in Gastric Cancer with Peritoneal Metastasis. Oncol. Lett..

[60] Saito H., Fushida S., Miyashita T., Oyama K., Yamaguchi T., Tsukada T., Kinoshita J., Tajima H., Ninomiya I., Ohta T. (2017). Potential of Extravasated Platelet Aggregation as a Surrogate Marker for Overall Survival in Patients with Advanced Gastric Cancer Treated with Preoperative Docetaxel, Cisplatin and S-1: A Retrospective Observational Study. BMC Cancer.

[61] Blair P., Flaumenhaft R. (2009). Platelet α-Granules: Basic Biology and Clinical Correlates. Blood Rev..

[62] Undas A., Ariëns R.A.S. (2011). Fibrin Clot Structure and Function: A Role in the Pathophysiology of Arterial and Venous Thromboembolic Diseases. Arterioscler. Thromb. Vasc. Biol..

[63] Pretorius E. (2007). The Role of Platelet and Fibrin Ultrastructure in Identifying Disease Patterns. Pathophysology Haemost. Thromb..

[64] Gomes F.G., Sandim V., Almeida V.H., Rondon A.M.R., Succar B.B., Hottz E.D., Leal A.C., Verçoza B.R.F., Rodrigues J.C.F., Bozza P.T. (2017). Breast-Cancer Extracellular Vesicles Induce Platelet Activation and Aggregation by Tissue Factor-Independent and -Dependent Mechanisms. Thromb. Res..

[65] Spillane C.D., Cooke N.M., Ward M.P., Kenny D., Blackshields G., Kelly T., Bates M., Huang Y., Martin C., Skehan S. (2021). The Induction of a Mesenchymal Phenotype by Platelet Cloaking of Cancer Cells Is a Universal Phenomenon. Transl. Oncol..

[66] Le Chapelain O., Ho-Tin-noé B. (2022). Intratumoral Platelets: Harmful or Incidental Bystanders of the Tumor Microenvironment?. Cancers.

[67] Li R., Ren M., Chen N., Luo M., Deng X., Xia J., Yu G., Liu J., He B., Zhang X. (2014). Presence of Intratumoral Platelets Is Associated with Tumor Vessel Structure and Metastasis. BMC Cancer.

[68] Obermann W.M.J., Brockhaus K., Eble J.A. (2021). Platelets, Constant and Cooperative Companions of Sessile and Disseminating Tumor Cells, Crucially Contribute to the Tumor Microenvironment. Front. Cell Dev. Biol..

[69] Mezouar S., Frère C., Darbousset R., Mege D., Crescence L., Dignat-george F., Panicot-dubois L., Dubois C. (2016). Role of Platelets in Cancer and Cancer-Associated Thrombosis: Experimental and Clinical Evidences. Thromb. Res..

[70] Ward Y., Lake R., Faraji F., Sperger J., Martin P., Gilliard C., Ku K.P., Rodems T., Niles D., Tillman H. (2018). Platelets Promote Metastasis via Binding Tumor CD97 Leading to Bidirectional Signaling That Coordinates Transendothelial Migration. Cell Rep..

[71] Li N. (2016). Platelets in Cancer Metastasis: To Help the “Villain” to Do Evil. Int. J. Cancer.

[72] Placke T., Kopp H.-G., Kanz L., Salih H.R. (2009). Coating of Tumor Cells by Platelets Confers Expression of Immunoregulatory Molecules which Impair NK Cell Anti-Tumor Reactivity. Blood.

[73] Raab S., Kropp K.N., Steinle A., Kanz L., Kopp H.-G., Salih H.R. (2013). Platelets Impair NK Cell Immunosurveillance of Metastasizing Tumor Cells by Altering Surface Expression and Shedding of Ligands for the Activating Immunoreceptor NKG2D. Blood.

[74] Clar K.L., Hinterleitner C., Schneider P., Salih H.R., Maurer S. (2019). Inhibition of NK Reactivity against Solid Tumors by Platelet-Derived RANKL. Cancers.

[75] Sadallah S., Schmied L., Eken C., Charoudeh H.N., Amicarella F., Schifferli J.A. (2016). Platelet-Derived Ectosomes Reduce NK Cell Function. J. Immunol..

[76] Schlesinger M. (2018). Role of Platelets and Platelet Receptors in Cancer Metastasis. J. Hematol. Oncol..

[77] Chen D., Abrahams J.M., Smith L.M., McVey J.H., Lechler R.I., Dorling A. (2008). Regenerative Repair after Endoluminal Injury in Mice with Specific Antagonism of Protease Activated Receptors on CD34^+^ Vascular Progenitors. Blood.

[78] Janowska-Wieczorek A., Wysoczynski M., Kijowski J., Marquez-Curtis L., Machalinski B., Ratajczak J., Ratajczak M.Z. (2005). Microvesicles Derived from Activated Platelets Induce Metastasis and Angiogenesis in Lung Cancer. Int. J. Cancer.

[79] Zarà M., Canobbio I., Visconte C., Canino J., Torti M., Guidetti G.F. (2018). Molecular Mechanisms of Platelet Activation and Aggregation Induced by Breast Cancer Cells. Cell. Signal..

[80] Augustine T.N., Pather K., Mak D., Klonaros D., Xulu K., Dix-Peek T., Duarte R., van der Spuy W.J. (2020). Ex Vivo Interaction between Blood Components and Hormone-Dependent Breast Cancer Cells Induces Alterations Associated with Epithelial-Mesenchymal Transition and Thrombosis. Ultrastruct. Pathol..

[81] Labelle M., Begum S., Hynes R.O. (2011). Platelets and Cancer Cells Induces an Epithelial-Mesenchymal-like Transition and Promotes Metastasis. Cancer Cell.

[82] Chang Y.I., Lee J.I.E.J., Chen W.E.I.F., Chou D.S., Huang S.Y., Sheu J.R. (2011). A Novel Role for Tamoxifen in the Inhibition of Human Platelets. Transl. Res..

[83] Johnson K.E., Forward J.A., Tippy M.D., Ceglowski J.R., El-Husayni S., Kulenthirarajan R., Machlus K.R., Mayer E.L., Italiano J.E., Battinelli E.M. (2017). Tamoxifen Directly Inhibits Platelet Angiogenic Potential and Platelet-Mediated Metastasis. Arterioscler. Thromb. Vasc. Biol..

[84] Pather K., Augustine T.N. (2022). Modelling the Procoagulatory Effect of Anastrozole Relative to ERα and ERβ Expression in Breast Cancer Cells. J. Thromb. Thrombolysis.

[85] Onitilo A.A., Doi S.A.R., Engel J.M., Glurich I., Johnson J., Berg R. (2012). Clustering of Venous Thrombosis Events at the Start of Tamoxifen Therapy in Breast Cancer: A Population-Based Experience. Thromb. Res..

[86] Asghar S., Parvaiz F., Manzoor S. (2019). Multifaceted Role of Cancer Educated Platelets in Survival of Cancer Cells. Thromb. Res..

[87] Wang L., Wang X., Guo E., Mao X., Miao S. (2022). Emerging Roles of Platelets in Cancer Biology and Their Potential as Therapeutic Targets. Front. Oncol..

[88] Tello-Montoliu A., Tomasello S.D., Ueno M., Angiolillo D.J. (2011). Antiplatelet Therapy: Thrombin Receptor Antagonists. Br. J. Clin. Pharmacol..

[89] Yun S.H., Sim E.H., Goh R.Y., Park J.I., Han J.Y. (2016). Platelet Activation: The Mechanisms and Potential Biomarkers. Biomed Res. Int..

[90] Osafo N., Agyare C., Obiri D.D., Antwi A.O., Al-Kaf A.G. (2017). Mechanism of Action of Nonsteroidal Anti-Inflammatory Drugs. Nonsteroidal Anti-Inflammatory Drugs.

[91] Cadavid A.P. (2017). Aspirin: The Mechanism of Action Revisited in the Context of Pregnancy Complications. Front. Immunol..

[92] Awtry E.H., Loscalzo J. (2000). Aspirin. Circulation.

[93] Bruno A., Dovizio M., Tacconelli S., Contursi A., Ballerini P., Patrignani P. (2018). Antithrombotic Agents and Cancer. Cancers.

[94] Rao P.P.N., Knaus E.E. (2008). Evolution of Nonsteroidal Anti-Inflammatory Drugs (NSAIDs): Cyclooxygenase (COX) Inhibition and Beyond. J. Pharm. Pharm. Sci..

[95] Reiter R., Resch U., Sinzinger H. (2001). Do Human Platelets Express COX-2?. Prostaglandins Leukot. Essent. Fat. Acids.

[96] Xu X.R., Yousef G.M., Ni H. (2018). Cancer and Platelet Crosstalk: Opportunities and Challenges of Aspirin and Other Antiplatelet Agents. Blood.

[97] Ferreiro J.L., Angiolillo D.J. (2012). New Directions in Antiplatelet Therapy. Circ. Cardiovasc. Interv..

[98] Wang D., Dubois R.N. (2006). Prostaglandins and Cancer. Gut.

[99] Hoellen F., Kelling K., Dittmer C., Diedrich K., Friedrich M., Thill M. (2011). Impact of Cyclooxygenase-2 in Breast Cancer. Anticancer Res..

[100] Kochel T.J., Goloubeva O.G., Fulton A.M. (2016). Upregulation of Cyclooxygenase-2/Prostaglandin E2 (COX-2/PGE2) Pathway Member Multiple Drug Resistance-Associated Protein 4 (MRP4) and Downregulation of Prostaglandin Transporter (PGT) and 15-Prostaglandin Dehydrogenase (15-PGDH) in Triple-Negative Breast. Breast Cancer Basic Clin. Res..

[101] Liu B., Qu L., Yan S. (2015). Cyclooxygenase-2 Promotes Tumor Growth and Suppresses Tumor Immunity. Cancer Cell Int..

[102] Bowers L.W., Maximo I.X.F., Brenner A.J., Beeram M., Hursting S.D., Price R.S., Tekmal R.R., Jolly C.A., DeGraffenried L.A. (2014). NSAID Use Reduces Breast Cancer Recurrence in Overweight and Obese Women: Role of Prostaglandin-Aromatase Interactions. Cancer Res..

[103] Jacobs E.J., Thun M.J., Bain E.B., Rodriguez C., Henley S.J., Calle E.E. (2007). A Large Cohort Study of Long-Term Daily Use of Adult-Strength Aspirin and Cancer Incidence. J. Natl. Cancer Inst..

[104] Jacobo-Herrera N.J., Pérez-Plasencia C., Camacho-Zavala E., Figueroa González G., López Urrutia E., Garćia-Castillo V., Zentella-Dehesa A. (2014). Clinical Evidence of the Relationship between Aspirin and Breast Cancer Risk (Review). Oncol. Rep..

[105] Lucotti S., Cerutti C., Soyer M., Gil-Bernabé A.M., Gomes A.L., Allen P.D., Smart S., Markelc B., Watson K., Armstrong P.C. (2019). Aspirin Blocks Formation of Metastatic Intravascular Niches by Inhibiting Platelet-Derived COX-1/Thromboxane A2. J. Clin. Investig..

[106] Frisk G., Ekberg S., Lidbrink E., Eloranta S., Sund M., Fredriksson I., Lambe M., Smedby K.E. (2018). No Association between Low-Dose Aspirin Use and Breast Cancer Outcomes Overall: A Swedish Population-Based Study. Breast Cancer Res..

[107] Loomans-Kropp H.A., Pinsky P., Umar A. (2021). Evaluation of Aspirin Use with Cancer Incidence and Survival among Older Adults in the Prostate, Lung, Colorectal, and Ovarian Cancer Screening Trial. JAMA Netw. Open.

[108] Bertrand K.A., Bethea T.N., Gerlovin H., Coogan P.F., Barber L., Rosenberg L., Palmer J.R. (2020). Aspirin Use and Risk of Breast Cancer in African American Women. Breast Cancer Res..

[109] Huang X.Z., Gao P., Sun J.X., Song Y.X., Tsai C.C., Liu J., Chen X.W., Chen P., Xu H.M., Wang Z.N. (2015). Aspirin and Nonsteroidal Anti-Inflammatory Drugs after but Not before Diagnosis Are Associated with Improved Breast Cancer Survival: A Meta-Analysis. Cancer Causes Control.

[110] Liu J., Zheng F., Yang M., Wu X., Liu A. (2021). Effect of Aspirin Use on Survival Benefits of Breast Cancer Patients. Medicine.

[111] McNeil J.J., Nelson M.R., Woods R.L., Lockery J.E., Wolfe R., Reid C.M., Kirpach B., Shah R.C., Ives D.G., Storey E. (2018). Effect of Aspirin on All-Cause Mortality in the Healthy Elderly. N. Engl. J. Med..

[112] Li H., Lee M.-H., Liu K., Wang T., Song M., Han Y., Yao K., Xie H., Zhu F., Grossmann M. (2017). Inhibiting Breast Cancer by Targeting the Thromboxane A2 Pathway. NPJ Precis. Oncol..

[113] Undas A., Brummel-Ziedins K.E., Mann K.G. (2007). Antithrombotic Properties of Aspirin and Resistance to Aspirin: Beyond Strictly Antiplatelet Actions. Blood.

[114] Lian L., Li W., Li Z.Y., Mao Y.X., Zhang Y.T., Zhao Y.M., Chen K., Duan W.M., Tao M. (2013). Inhibition of MCF-7 Breast Cancer Cell-Induced Platelet Aggregation Using a Combination of Antiplatelet Drugs. Oncol. Lett..

[115] Johnson K.E., Ceglowski J.R., Roweth H.G., Forward J.A., Tippy M.D., El-Husayni S., Kulenthirarajan R., Malloy M.W., Machlus K.R., Chen W.Y. (2019). Aspirin Inhibits Platelets from Reprogramming Breast Tumor Cells and Promoting Metastasis. Blood Adv..

[116] Passacquale G., Ferro A. (2011). Current Concepts of Platelet Activation: Possibilities for Therapeutic Modulation of Heterotypic vs. Homotypic Aggregation. Br. J. Clin. Pharmacol..

[117] Eikelboom J.W., Hirsh J., Spencer F.A., Baglin T.P., Weitz J.I. (2012). Antiplatelet Drugs—Antithrombotic Therapy and Prevention of Thrombosis, 9th Ed: American College of Chest Physicians Evidence-Based Clinical Practice Guidelines. Chest.

[118] Damman P., Woudstra P., Kuijt W.J., De Winter R.J., James S.K. (2012). P2Y12 Platelet Inhibition in Clinical Practice. J. Thromb. Thrombolysis.

[119] Ballerini P., Dovizio M., Bruno A., Tacconelli S., Patrignani P. (2018). P2Y 12 Receptors in Tumorigenesis and Metastasis. Front. Pharmacol..

[120] Denslow A., Świtalska M., Jarosz J., Papiernik D., Porshneva K., Nowak M., Wietrzyk J. (2017). Clopidogrel in a Combined Therapy with Anticancer Drugs—Effect on Tumor Growth, Metastasis, and Treatment Toxicity: Studies in Animal Models. PLoS ONE.

[121] Burnstock G. (2017). Purinergic Signaling in the Cardiovascular System. Circ. Res..

[122] Yi X., Lin J., Wang Y., Zhou Q., Wang C., Cheng W., Chi L. (2016). Association of Cytochrome P450 Genetic Variants with Clopidogrel Resistance and Outcomes in Acute Ischemic Stroke. J. Atheroscler. Thromb..

[123] Maguire A., Douglas I., Smeeth L., Thompson M. (2007). Determinants of Cholesterol and Triglycerides Recording in Patients Treated with Lipid Lowering Therapy in UK Primary Care. Pharmacoepidemiol. Drug Saf..

[124] Roden D.M., Stein C.M. (2009). Clopidogrel and the Concept of High-Risk Pharmacokinetics. Circulation.

[125] Sangkuhl K., Klein T.E., Altman R.B. (2010). Clopidogrel Pathway. Pharmacogenet. Genom..

[126] Elmariah S., Doros G., Benavente O.R., Bhatt D.L., Connolly S.J., Yusuf S., Steinhubl S.R., Liu Y., Hsieh W.H., Yeh R.W. (2018). Impact of Clopidogrel Therapy on Mortality and Cancer in Patients with Cardiovascular and Cerebrovascular Disease: A Patient-Level Meta-Analysis. Circ. Cardiovasc. Interv..

[127] Mauri L., Kereiakes D.J., Yeh R.W., Driscoll-Shempp P., Cutlip D.E., Steg P.G., Normand S.T., Wiviott S.D., Cohen D.J., Holmes D.R. (2014). Twelve or 30 Months of Dual Antiplatelet Therapy After Drug-Eluting Stents. N. Engl. J. Med..

[128] Capodanno D., Ferreiro J.L., Angiolillo D.J. (2013). Antiplatelet Therapy: New Pharmacological Agents and Changing Paradigms. J. Thromb. Haemost..

[129] Roop R.P., Naughton M.J., Van Poznak C., Schneider J.G., Lammers P.E., Pluard T.J., Johnson F., Eby C.S., Weilbaecher K.N. (2013). A Randomized Phase Ii Trial Investigating the Effect of Platelet Function Inhibition on Circulating Tumor Cells in Patients with Metastatic Breast Cancer. Clin. Breast Cancer.

[130] Roe M.T., Cyr D.D., Eckart D., Schulte P.J., Morse M.A., Blackwell K.L., Ready N.E., Zafar S.Y., Beaven A.W., Strickler J.H. (2016). Ascertainment, Classification, and Impact of Neoplasm Detection during Prolonged Treatment with Dual Antiplatelet Therapy with Prasugrel vs. Clopidogrel Following Acute Coronary Syndrome. Eur. Heart J..

[131] Serebruany V.L., Cherepanov V., Cabrera-Fuentes H.A., Kim M.H. (2015). Solid Cancers after Antiplatelet Therapy: Confirmations, Controversies, and Challenges. Thromb. Haemost..

[132] Yusuf S., Zhao F., Mehta S., Chrolavicius S., Tognoni K., Fox K. (2001). Clopidogrel in Unstable Angina to Prevent Recurrent Events Trial Investigators Effects of Clopidogrel in Addition to Aspirin in Patients with Acute Coronary Syndromes without ST-Segment Elevation. N. Engl. J. Med..

[133] Active Investigators (2009). Effect of Clopidogrel Added to Aspirin in Patients with Atrial Fibrillation. N. Engl. J. Med..

[134] Leader A., Zelikson-Saporta R., Pereg D., Spectre G., Rozovski U., Raanani P., Hermoni D., Lishner M. (2017). The Effect of Combined Aspirin and Clopidogrel Treatment on Cancer Incidence. Am. J. Med..

[135] Mauri L., Elmariah S., Yeh R.W., Cutlip D.E., Steg P.G., Windecker S., Wiviott S.D., Cohen D.J., Massaro J.M., D’Agostino R.B. (2016). Causes of Late Mortality with Dual Antiplatelet Therapy after Coronary Stents. Eur. Heart J..

[136] Wiviott S.D., Braunwald E., McCabe C., Giles Montalescot B., Ruzyllo W., Gottlieb S., Neumann F.J., Ardissino D., De Servi S., Murphy S. (2007). Prasugrel versus Clopidogrel in Patients with Acute Coronary Syndromes. N. Engl. J. Med..

[137] Smeda M., Kieronska A., Proniewski B., Jasztal A., Selmi A., Wandzel K., Zakrzewska A., Wojcik T., Przyborowski K., Derszniak K. (2018). Dual Antiplatelet Therapy with Clopidogrel and Aspirin Increases Mortality in 4T1 Metastatic Breast Cancer-Bearing Mice by Inducing Vascular Mimicry in Primary Tumour. Oncotarget.

[138] Xulu K., Duarte R., Augustine T. (2020). Combined Anastrozole and Antiplatelet Therapy Treatment Differentially Promotes Breast Cancer Cell Survival. Microsc. Microanal..

[139] Jennings L.K. (2009). Mechanisms of Platelet Activation: Need for New Strategies to Protect against Platelet-Mediated Atherothrombosis. Thromb. Haemost..

[140] Wojtukiewicz M.Z., Hempel D., Sierko E., Tucker S.C., Honn K.V. (2016). Thrombin—Unique Coagulation System Protein with Multifaceted Impacts on Cancer and Metastasis. Cancer Metastasis Rev..

[141] Heuberger D.M., Schuepbach R.A. (2019). Correction to: Protease-Activated Receptors (PARs): Mechanisms of Action and Potential Therapeutic Modulators in PAR-Driven Inflammatory Diseases. Thromb. J..

[142] Mihara K., Ramachandran R., Saifeddine M., Hansen K.K., Renaux B., Polley D., Gibson S., Vanderboor C., Hollenberg M.D. (2016). Thrombin-Mediated Direct Activation of Proteinase-Activated Receptor-2: Another Target for Thrombin Signaling. Mol. Pharmacol..

[143] Holinstat M., Preininger A.M., Milne S.B., Hudson W.J., Brown H.A., Hamm H.E. (2009). Irreversible Platelet Activation Requires Protease-Activated Receptor 1-Mediated Signaling to Phosphatidylinositol Phosphates. Mol. Pharmacol..

[144] Tourdot B.E., Stoveken H., Trumbo D., Yeung J., Kanthi Y., Edelstein L.C., Bray P.F., Tall G.G., Holinstat M. (2018). Genetic Variant in Human PAR (Protease-Activated Receptor) 4 Enhances Thrombus Formation Resulting in Resistance to Antiplatelet Therapeutics. Arterioscler. Thromb. Vasc. Biol..

[145] Han N., Jin K., He K., Cao J., Teng L. (2011). Protease-Activated Receptors in Cancer: A Systematic Review. Oncol. Lett..

[146] Schuepbach R.A., Madon J., Ender M., Galli P., Riewald M. (2012). Protease-Activated Receptor-1 Cleaved at R46 Mediates Cytoprotective Effects. J. Thromb. Haemost..

[147] Reddel C.J., Tan C.W., Chen V.M. (2019). Thrombin Generation and Cancer: Contributors and Consequences. Cancers.

[148] Ay C., Dunkler D., Simanek R., Thaler J., Koder S., Marosi C., Zielinski C., Pabinger I. (2011). Prediction of Venous Thromboembolism in Patients with Cancer by Measuring Thrombin Generation: Results from the Vienna Cancer and Thrombosis Study. J. Clin. Oncol..

[149] Renni M.J.P., Cerqueira M.H., de Araújo Trugilho I., Araujo Junior M.L.C., Marques M.A., Koch H.A. (2017). Mechanisms of Venous Thromboembolism in Cancer: A Literature Review. J. Vasc. Bras..

[150] Covic L., Kuliopulos A. (2018). Protease-Activated Receptor 1 as Therapeutic Target in Breast, Lung, and Ovarian Cancer: Pepducin Approach. Int. J. Mol. Sci..

[151] Wang Y., Liao R., Chen X., Ying X., Chen G., Li M., Dong C. (2020). Twist-Mediated PAR1 Induction Is Required for Breast Cancer Progression and Metastasis by Inhibiting Hippo Pathway. Cell Death Dis..

[152] Schweickert P.G., Yang Y., White E.E., Cresswell G.M., Elzey B.D., Ratliff T.L., Arumugam P., Antoniak S., Mackman N., Flick M.J. (2021). Thrombin-PAR1 Signaling in Pancreatic Cancer Promotes an Immunosuppressive Microenvironment. J. Thromb. Haemost..

[153] Moschonas I.C., Goudevenos J.A., Tselepis A.D. (2015). Protease-Activated Receptor-1 Antagonists in Long-Term Antiplatelet Therapy. Current State of Evidence and Future Perspectives. Int. J. Cardiol..

[154] Kogushi M., Matsuoka T., Kawata T., Kuramochi H., Kawaguchi S., Murakami K., Hiyoshi H., Suzuki S., Kawahara T., Kajiwara A. (2011). The Novel and Orally Active Thrombin Receptor Antagonist E5555 (Atopaxar) Inhibits Arterial Thrombosis without Affecting Bleeding Time in Guinea Pigs. Eur. J. Pharmacol..

[155] Xiang Q., Pang X., Liu Z., Yang G., Tao W., Pei Q., Cui Y. (2019). Progress in the Development of Antiplatelet Agents: Focus on the Targeted Molecular Pathway from Bench to Clinic. Pharmacol. Ther..

[156] Wiviott S.D., Flather M.D., O’Donoghue M.L., Goto S., Fitzgerald D.J., Cura F., Aylward P., Guetta V., Dudek D., Contant C.F. (2011). Randomized Trial of Atopaxar in the Treatment of Patients with Coronary Artery Disease: The Lessons from Antagonizing the Cellular Effect of Thrombin–Coronary Artery Disease Trial. Circulation.

[157] Goto S., Ogawa H., Takeuchi M., Flather M.D. (2010). Double-Blind, Placebo-Controlled Phase II Studies of the Protease-Activated Receptor 1 Antagonist E5555 (Atopaxar) in Japanese Patients with Acute Coronary Syndrome or High-Risk Coronary Artery Disease. Eur. Heart J..

[158] Liu X., Yu J., Song S., Yue X., Li Q. (2017). Protease-Activated Receptor-1 (PAR-1): A Promising Molecular Target for Cancer. Oncotarget.

[159] Yang E., Boire A., Agarwal A., Nguyen N., O’Callaghan K., Tu P., Kuliopulos A., Covic L. (2009). Blockade of PAR1 Signaling with Cell-Penetrating Pepducins Inhibits Akt Survival Pathways in Breast Cancer Cells and Suppresses Tumor Survival and Metastasis. Cancer Res..

[160] Wang Y., Liu J., Ying X., Lin P.C., Zhou B.P. (2016). Twist-Mediated Epithelial-Mesenchymal Transition Promotes Breast Tumor Cell Invasion via Inhibition of Hippo Pathway. Sci. Rep..

[161] Sun J., Du Y., Zhang X., Wang Z., Lin Y., Song Q., Wang X., Guo J., Li S., Nan J. (2019). Discovery and Evaluation of Atopaxar Hydrobromide, a Novel JAK1 and JAK2 Inhibitor, Selectively Induces Apoptosis of Cancer Cells with Constitutively Activated STAT3. Investig. New Drugs.

